# Can walking capacity predict respiratory functions of people with Parkinson’s disease?

**DOI:** 10.3389/fneur.2025.1531571

**Published:** 2025-03-26

**Authors:** Lucas Meireles Matos, Francisca Maria de Araujo Oliveira, Rodrigo Santiago Barbosa Rocha, Aline da Silva Pimentel, Laura Maria Tomazi Neves, Alex Harley Crisp, Leonardo Alexandre Peyré-Tartaruga, Luca Correale, Marcelo Coertjens, Elren Passos-Monteiro

**Affiliations:** ^1^Graduate Program in Human Movement Sciences, Federal University of Pará, Belém, Brazil; ^2^Degree in Physical Education, Federal University of Pará, Castanhal, Brazil; ^3^Department of Human Movement Sciences, State University of Pará, Belém, Brazil; ^4^Human Locomotion Laboratory (LocoLab), Department of Public Health, Experimental and Forensic Medicine, University of Pavia, Pavia, Italy; ^5^LaBiodin Biodynamics Laboratory, Universidade Federal do Rio Grande do Sul, Porto Alegre, Brazil; ^6^Graduate Program in Biomedical Sciences, Federal University of Delta do Parnaíba, Parnaíba, Brazil

**Keywords:** movement disorder, locomotion, motor rehabilitation, lung test, respiratory disease, Parkinson’s disease

## Abstract

**Introduction:**

People with Parkinson’s Disease (PwPD) and an impaired respiratory profile show a lower walking ability. Still, it is unknown if there is a relationship between walking ability and respiratory function that can be used to predict the latter. This cross-sectional study evaluated the relationship between walking ability and respiratory function in PwPD.

**Methods:**

Seventeen older PwPD, between 60 and 80 years old were asked to perform a 10-m walking test at self-selected, fast speed, and respiratory tests and these variables analyzed by an multiple linear regression.

**Results:**

The respiratory profile revealed that 44% of the patients were restrictive, 33% were obstructive, and 22% were mixed. 73% of the PwPD presented a low lung capacity, as demonstrated by the forced expiratory volume in 1 s divided by the forced vital capacity (FEV1/FVC). Multiple linear regression demonstrated that self-selected walking speed explained 53 and 58% (*p* = 0.027 and *p* = 0.016) of the variation in maximal inspiratory and expiratory pressures, respectively. The fast walking speed explained 62 and 66% (*p* = 0.008 and *p* = 0.005) of the maximal inspiratory and expiratory pressure variation, respectively. Furthermore, the locomotor rehabilitation index explained 39% (*p* = 0.022) of the variance in the FEV1/FVC.

**Conclusion:**

These results suggest that walking ability, particularly at self-selected and fast speeds, is a suitable screening parameter for pulmonary impairments in PwPD. Furthermore, the locomotor rehabilitation index indicates the ability to expire rapidly as a proportion of forced vital capacity in PwPD. Thus, the walking ability test can be an easily applicable and low-cost biomarker for assessing respiratory changes in PwPD.

## Introduction

People with Parkinson’s disease (PwPD) often exhibit difficulty walking, characterized by short, slow steps, increased time of foot contact with the ground, and reduced angles of lower limb flexion (1–3). Due to these alterations, self-selected walking speed (SSWS) is reduced, requiring greater motor planning (4) and increased metabolic cost (5, 6). The reduced gait speed in PwPD may be explained by the reduced conversion of potential and kinetic energies, resulting in decreased optimization of the pendulum-like mechanism (6, 7). The increased metabolic cost due to an impaired pendulum-like mechanism may imply increased respiratory demand.

In PwPD, the main modification in pulmonary function is characterized by a restrictive disorder, with reduced inspired air volume and decreased muscle inspiratory (MIP) and muscle expiratory (MEP) pressures (8, 9). Although respiratory changes are underdiagnosed, they present with disease progression and accompanying motor and autonomic changes in PwPD. Furthermore, these changes involve reduced respiratory muscle strength and abnormalities in abdominal and diaphragmatic coordination, which consequently lead to impaired swallowing, persistent cough, dyspnea, and limitations in ventilatory capacity and lung compliance (10–12).

In respiratory and cardiac diseases, the relationship between functional mobility and respiratory function is well-established (13–15). Despite these findings in the literature, the question of whether alterations in gait capacity can predict respiratory changes in PwPD is unknown. Thus, we aimed to analyze the respiratory profile of PwPD and further investigate whether gait capacity is associated with respiratory changes in PwPD. We hypothesize that PwPD has respiratory and gait capacity alterations and that low walking speed and a lower locomotor rehabilitation index (LRI) may predict respiratory muscle weakness in PwPD. Our hypothesis is based on previous findings that impaired respiratory parameters are associated with reduced walking ability in restrictive (14) and obstructive pulmonary diseases (15).

## Methods

### Study design

This study has a cross-sectional design, described according to the Strengthening the Reporting of Observational Studies in Epidemiology (STROBE) checklist (36). The research was approved by the Research Ethics Committee of the Institute of Health Sciences of the Federal University of Pará (UFPA), Belém, Pará, Brazil, by the terms of Resolutions 466/2012 and 580/16 of the National Health Council, under protocol number CAAE 67654523.7.0000.0018 and registration by *Clinical Trials* by the number NCT04135924. The study was conducted at the Multidisciplinary Laboratory of Human Movement Analysis, Exercise, and Rehabilitation (LABMOVHER) of the UFPA.

### Participants

The study included elderly individuals between 60 and 80 who were clinically diagnosed with PD based on the presentation of a medical report at the initial interview, according to the UK Parkinson’s Disease Society Brain Bank Clinical Diagnostic Criteria (16).

For sample size calculation, Gpower® software (v.3.1.9.7, University of Kiel, Germany) was used with a power of 0.80, margin of error of 0.10, and significance level = 0.05, which was based on the means and standard deviations of the variables of the MIP from Ferro et al. (17), totaling *n* = 17. Additionally, a 20% loss of the sample size was considered according to the eligibility criteria. Thus, 36 individuals were recruited, and ultimately, 17 individuals were analyzed.

### Sample recruitment

Participants were recruited through printed materials in public places, social media announcements, and invitations to volunteers from a locomotor rehabilitation program for people with Parkinson’s disease (INSPIRE PARKINSON), as well as through printed materials in public places, health units, and invitations via social media. Recruitment occurred at LABMOVHER-UFPA.

### Eligibility criteria

As eligibility criteria for selection, volunteers were required to (1) be receiving levodopa therapy, (2) be classified by the modified Hoehn and Yahr (H&Y) scale between stages 2 and 4 of the disease, (3) have preserved cognition, assessed by the Mini-Mental State Examination (MMSE) with a score of 23 points or higher (18), and (4) have stable medication for at least the past 4 weeks. Before the initial interview, the participants read and signed the informed consent form. Individuals who were active alcohol drinkers or smokers or who were in the postoperative period for at least 6 months were not selected. Additionally, those who had undergone other types of treatment, such as deep brain stimulation (DBS), beta-blockers, sedatives, hypnotics, antibiotics, and anti-inflammatories, interfere with pulmonary function analysis. Participants who missed the evaluation day or could not complete the tests were excluded from the sample (19, 20).

### Data collection

Gender, age (years), body mass (kg), height (m), and body mass index (kg/m^2^) were used for spirometry, and lower limb length (m) was used to determine the optimal walking speed (21). Motor assessment and classification of PD were performed via the Movement Disorders Society-Unified Parkinson’s Disease Rating Scale (MDS-UPDRS) part III (19). The modified H&Y scale was subsequently used to classify the disease stage of each individual and the MMSE score according to their cognition.

### Primary outcome

Primary outcomes were measured by pulmonary function evaluated by spirometry, considering forced vital capacity (FVC) and forced expiratory volume in 1 s (FEV1) and its ratio of FEV1/FVC for respiratory profile classification, as well as respiratory muscle strength, expressed by maximal inspiratory and expiratory pressures, MIP, and MEP, respectively.

### Secondary outcomes

The secondary variables include specific characteristics of gait capacity: SSWS, OWS, fast walking speed (FWS), and LRI.

### Data collection procedure

Volunteers were invited to be assessed on 2 days, initially through anamnesis, clinical evaluation of Parkinson’s disease, and pulmonary function testing, followed by walking tests on the second day, all while in the ON state of medication, which was considered up to 3 h after ingestion (21). The participants with flu-like symptoms were required to return after 10 days for reassessment. Finally, pulmonary function and muscle inspiratory and expiratory pressure were assessed (for details from data collection and data processing, see Supplementary material 1) (22–24). Volunteers with flu-like symptoms were instructed to return for evaluation after 10 days for respiratory and motor tests.

### Gait capacity

Locomotor outcomes were assessed via the ten-meter walk test (10MWT) (25, 26). The SSWS is the speed at which an individual can typically walk in daily activities. The FWS is considered the maximum speed the individual reaches within 10 m. The optimal walking speed (OWS) was estimated utilizing the Froude constant (Fr) estimated at 0.25, the acceleration due to gravity (g), the length of the dominant lower limb, with the subject in an upright bipedal position, and measurements taken from the trochanter to the ground, as follows (Equation 1) (5, 21, 27).


(1) OWS=√0.25x9.81xCMI

The locomotor rehabilitation index (LRI) is a method used to determine how far the SSWS is from the OWS. It is an index that indicates the ability to walk efficiently and has some critical clinical applications (5, 26, 28). PwPD aims to bring the SSWS closer to the OWS to make walking more economical. We calculated using the following equation (Equation 2):


(2) LRI=100×SSWS/OWS

### Data analysis

#### Gait capacity

Gait capacity was determined by the analysis of the SWSS and FWS (km.h^−1^). The ten-meter walk test (10MWT) was conducted in a flat area totaling 14 meters, with the start and end of the course marked by cones. The participants walked at their self-selected speed, and the examiner timed their performance over the intermediate 10 meters, excluding the initial and final deceleration phases, marked by 2 meters (Supplementary material 2). Three tests were performed to minimize the learning effect, and the best performance was considered for data analysis (25, 26).

### Statistical analysis

We tested the normality of the data distribution through the Shapiro–Wilk test on the study’s outcomes. The product–moment Pearson test was applied to evaluate the correlations between walking and respiratory outcomes. A linear regression model was used for prediction between the evaluated variables, adjusted by the clinical covariate of staging and the condition of the modified Hoehn and Yahr disease. Additionally, Cook’s distance test was used to analyze the differences between the samples. A significance level for statistical inferences of *p* ≤ 0.05 and a 95% confidence interval (CI) was adopted. We used Rstudios software (4.3.0) for data processing and statistical analysis.

## Results

### Sample profile

**Figure 1:**
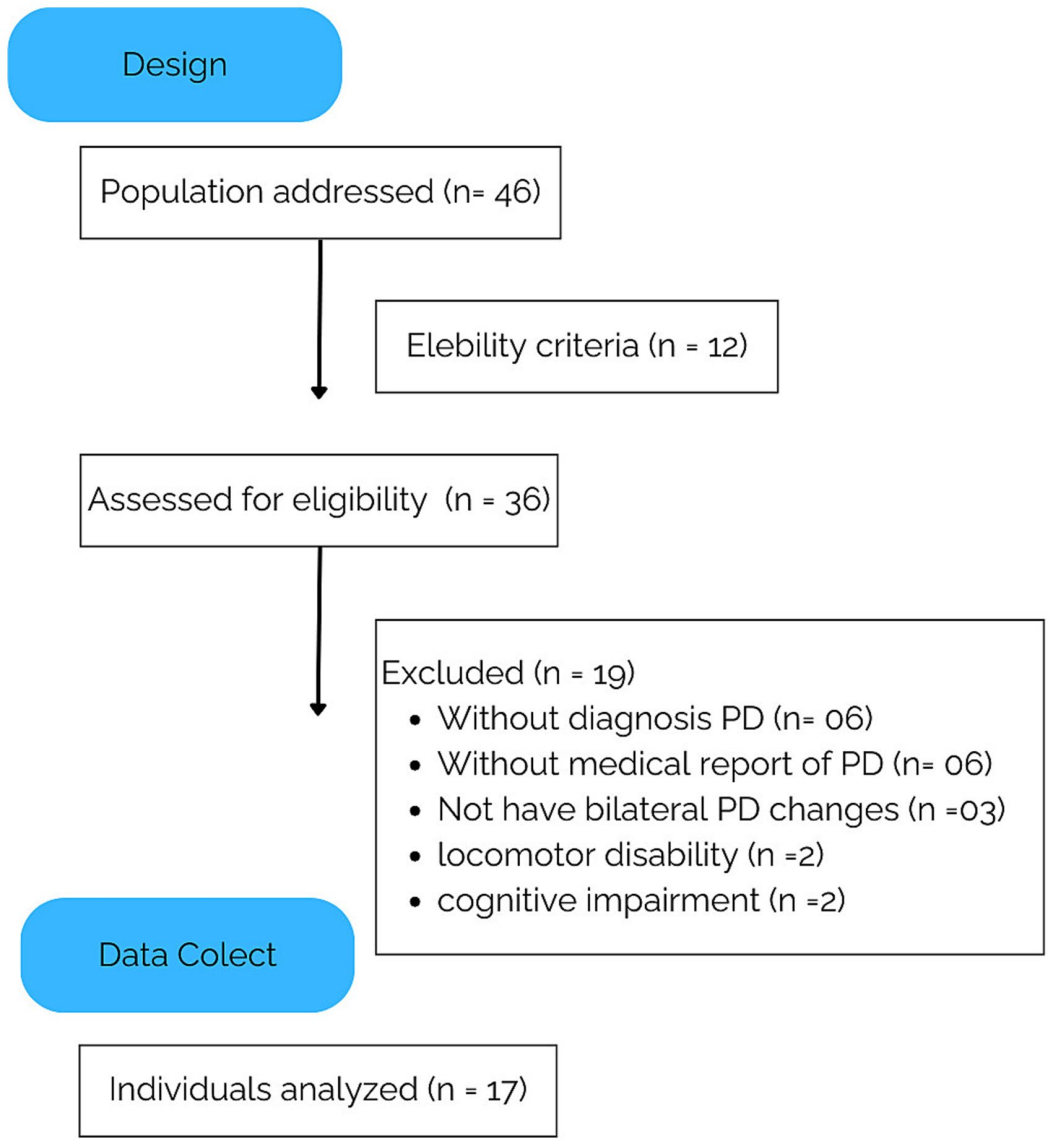
A flowchart about the individuals with Parkinson’s disease.

A total of 36 elderly PwPD participated in the initial sample. Those who did not have PD or did not present a medical report were excluded (*n* = 6), as were those who did not have PD (*n* = 6) and those who did not have bilateral PD changes (*n* = 3); furthermore, individuals with locomotor disabilities that impeded the assessment of gait capacity (*n* = 2) and those with cognitive impairments (*n* = 2) were excluded. After this eligibility criterion was met, 17 individuals were included in the respiratory profile analysis (the flowchart is shown in Figure 1).

The sample characterization data, as well as the clinical data, are described in Table 1.

**Table 1:** Mean, median, and standard deviation data for sample characterization (*n* = 17).

		95% Confidence interval		
	Mean	Lower limit	Upper limit	Sd (±)
Age (years)	66.59	62.30	70.88	8.34
H&Y*	2.5	2	4	0.50
UPDRS-MDS	29.76	24.06	35.47	11.09
Weight (kg)	66.92	59.34	74.50	14.22
Height (cm)	162.06	157.54	166.58	8.78
MMSE	24.53	22.87	26.19	2.99

H&Y is characterized as a categorical variable and its central tendency is represented by median and standard error; *Shapiro–Wilk normality test *p* < 0.05. Non-parametric data.

### Respiratory profile

**Figure 2:**
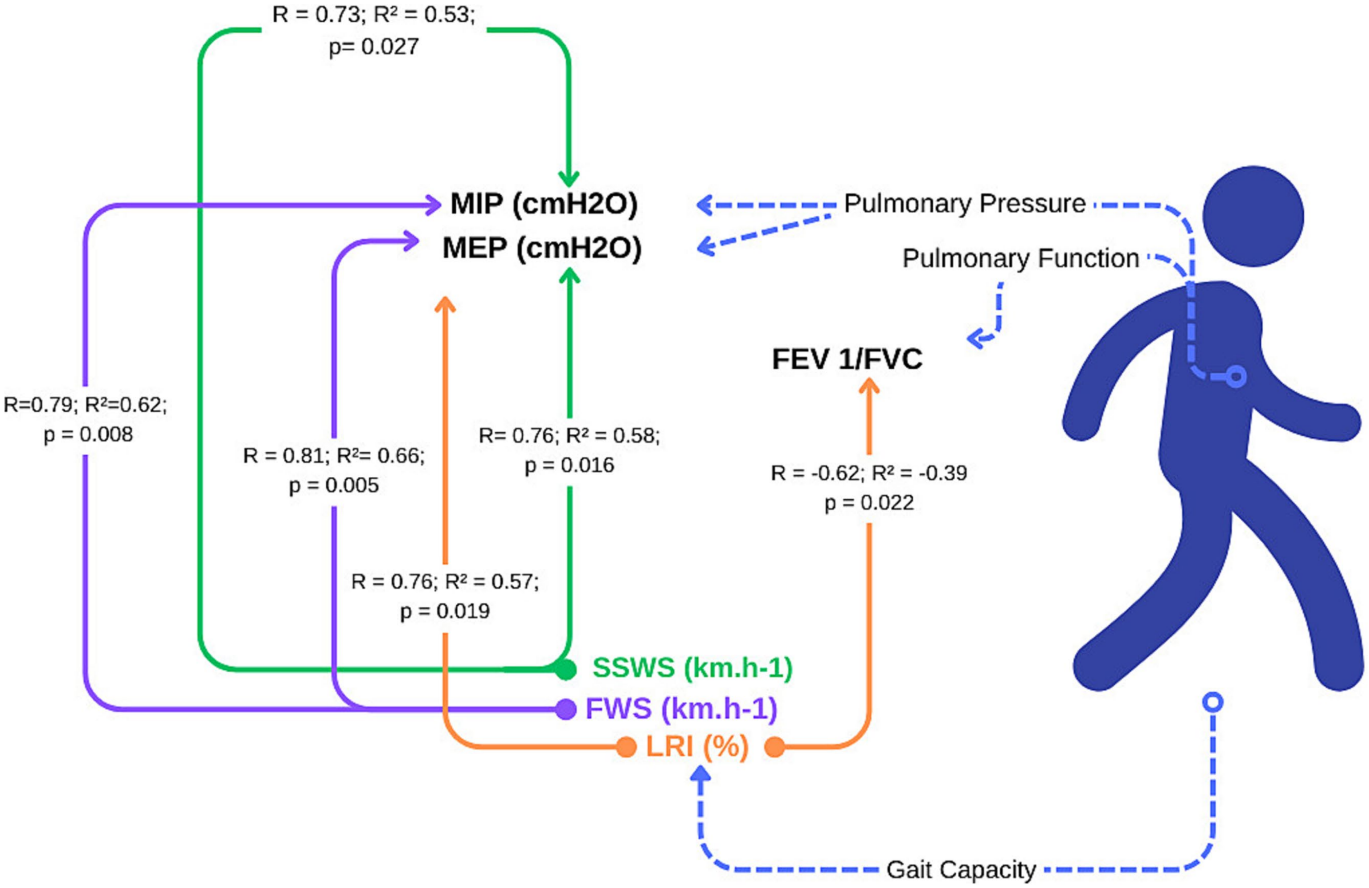
Prediction of respiratory function from gait capacity in individuals with Parkinson’s disease.

In the analysis of respiratory muscle strength (MIP and MEP), on average, PwPD exhibited a degree of respiratory muscle weakness compared with their predicted values (predicted MIP, predicted MEP). Furthermore, the results of FVC (predicted FVC) and FEV1 (predicted FEV1) also showed lower values, as described in Table 2. Concerning the respiratory profile classified by the ratio of FEV1/FVC, individuals exhibited normal, restrictive, obstructive, or mixed patterns. In terms of respiratory profile analysis, 52.9% of the samples exhibited some respiratory impairment (*n* = 09), with the restrictive profile being the most frequent among subgroups with any impairment, accounting for 44.4% (*n* = 4) of the samples, followed by the obstructive profile at 33.3% (*n* = 3) and the mixed profile at 22.2% (*n* = 2). Individuals without any respiratory impairment accounted for 47.06% (*n* = 8) of the sample (Figure 2).

**Table 2:** Description of respiratory and gait capacity variables of PwPD (*n* = 17).

			Confidence interval 95%		
	Mean	Sd	Lower limit	Upper limit	*p-*value
Respiratory variables					
MIP (cmH2O)	58.56	24.56	45.47	71.65	<0.01
MIPpred (cmH2O)	96.61	13.99	89.15	104.06	
%pred	59.88	22.15	48.07	71.68	
MEP (cmH2O)	80.81	31.86	63.83	97.79	<0.01
MEPpred (cmH2O)	102.84	19.27	92.58	113.11	
%pred	78.87	31.43	62.12	95.62	
FVC (l)	2.85	1.03	2.29	3.4	0.02
FVCpred (l)	3.47	0.76	3.06	3.87	
%FVC	79.08	16.2	70.44	87.72	
FEV1 (l)	2.07	0.74	1.68	2.47	<0.01
FEV1.pred (l)	2.67	0.6	2.34	2.99	
% pred	76.77	19.06	66.61	86.93	
FEV1/FVC (%)	72.53	10.12	67.13	77.92	0.03
FEV1/FVC.pred (%)	78.75	1.69	77.85	79.65	
PEF (l/min)	3.24	1.51	2.43	4.05	<0.01
PEF.pred (l/min)	9.09	1.98	8.03	10.15	
%pred	35.63	14.98	27.65	43.16	
Gait capacity					
SSWS (km.h-1)	3.99	1.04	3.42	4.53	
FWS (km.h-1)[S1]	4.75	1.22	4.11	5.36	
OWS	5.00	0.75	4.57	5.49	
LRI (%)	84.34	11.63	78.36	90.34	
Distance (m)	414.46	57.24	371.03	441.33	

Volunteers exhibited values below the predicted. Considering values below normality for age, sex, and BMI (kg/m^2^). *Indicates variables with non-normal distribution. The *p*-value demonstrates the difference between the predicted value and the obtained value in the respiratory analysis of the volunteers.

### Gait capacity profile

Thus, individuals presented a lower mean speed in SWSS (3.96 ± 1.04 km.h^−1^) than in the mean FWS (5.00 ± 0.75 km.h^−1^), resulting in an LRI of 84.3%. The locomotor data are presented in Table 2.

**Figure 3:**
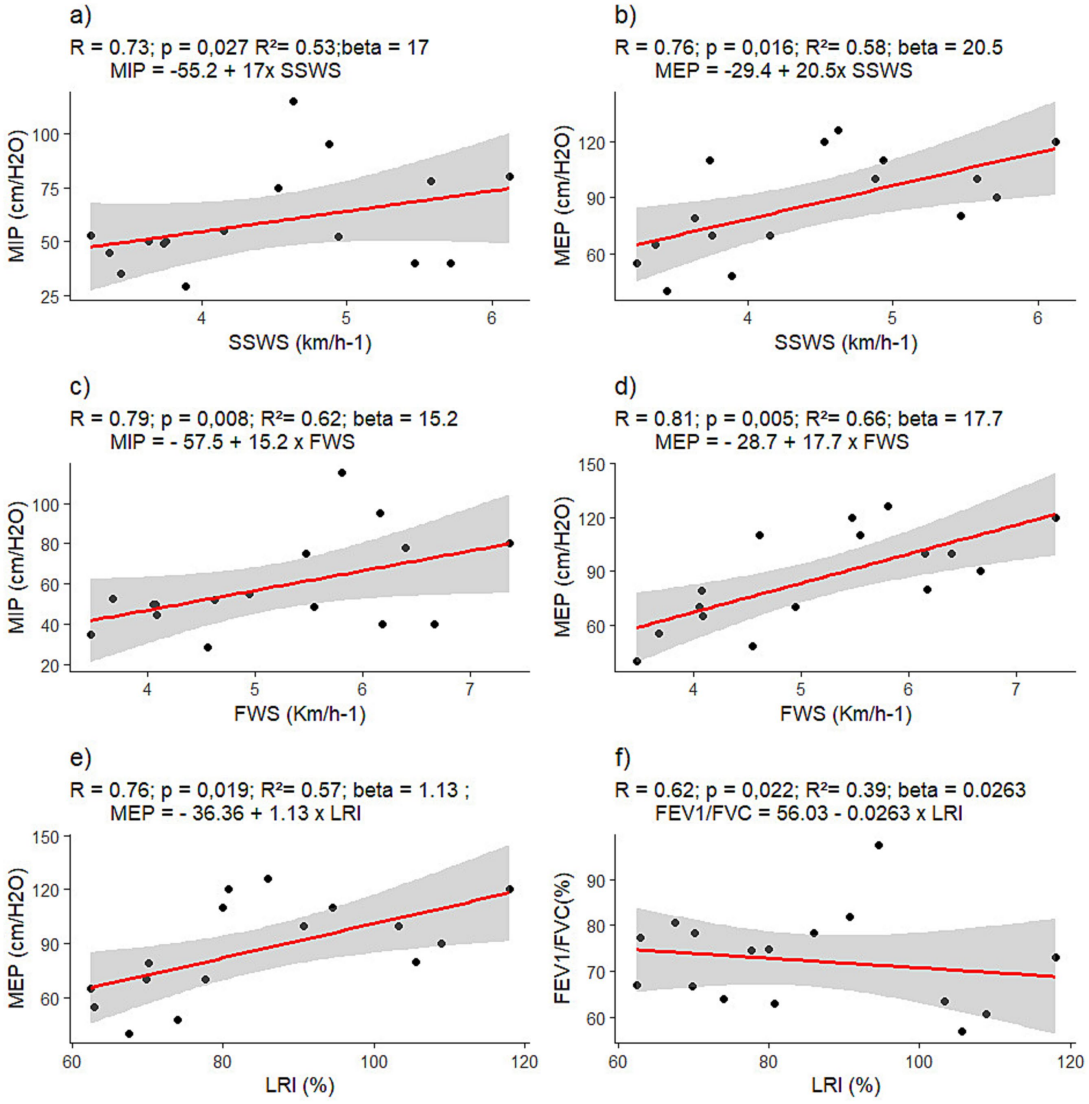
Model for the prediction of lung strength and function by gait capacity. **(A)** represents the relationship between MIP and SSWS, demonstrating a positive relationship, as well as figure **(B)** which demonstrates the relationship between MEP and SSWS, illustrating the interference of respiratory activity in walking capacity. In **(C, D)** are the relationship between FS and respiratory activity and the variation for each increase in speed (km.h^-1^) and in **(E, F)** the respiratory relationship and LRI, which in **(F)** demonstrates a negative relationship.

It is possible to observe the prediction for the analyzed outcomes, where we have demonstrated that for every 0.1 km.h^−1^ variation, there is a corresponding variation value (beta), as shown in Figure 3.

## Discussion

We have evaluated the relationship between walking ability and respiratory function in PwPD. Our findings demonstrate that gait capacity can predict respiratory changes, confirming the hypothesis that slow walking speed and lower LRIs predict respiratory muscle weakness in individuals with moderate PD. We observed that the SSWS explained 53 and 58% of the variation in respiratory muscle strength (MIP and MEP), respectively. Thus, gait capacity, particularly at self-selected and fast speeds, is a suitable screening parameter for pulmonary impairments.

The subjects in this study presented an average speed of 3.99 km.h-1, below the expected 5 km.h-1, with an LRI of 84.35%. In PwPD, walking ability is linked to mobility, independence in daily activities, and comorbidities (29). There is an optimal speed where the metabolic cost is lowest, and deviations increase cost, especially in Parkinson’s disease (5, 27). Thoracic mobility restrictions, postural changes, and rigidity contribute to more significant respiratory effort. The LRI indicates the ability to expire rapidly as a proportion of forced vital capacity in PwPD.

Additionally, 44% of the subjects had a restrictive profile, 33% had an obstructive profile, and 22% had a mixed profile. PwPD with bilateral impairment (H&Y > 2) presented a reduced walking speed and a restrictive, mixed, or obstructive respiratory pattern. Only eight participants had a normal respiratory pattern. The SSWS attained by our subjects was greater than those of Zanardi et al. (2) and Monteiro et al. (27). Our individuals have a more active lifestyle than in these previous studies, and some are Nordic walking practitioners. Interestingly, our findings show that walking ability in active PwPD may predict respiratory function. Motor coordination and respiration in PD patients are complex due to rigidity, bradykinesia, and abnormal posture (kyphosis), which affect respiratory pressure and pulmonary mechanics. Dopaminergic dysfunction impacts brainstem respiratory centers, leading to inadequate ventilation and increased respiratory risk (22, 30).

The degradation of the substantia nigra is associated with early changes in basal ganglia nuclei, leading to increased inflammation as the disease progresses (31). Peripheral regions, such as the striatum (including the putamen and caudate nucleus), part of the extrapyramidal system, are impacted by reduced dopaminergic projections from the substantia nigra. These regions are critical in voluntary movement regulation, including respiratory function. A decrease in dopamine within these regions disrupts excitatory circuits in the pons and medulla, particularly the pre-Bötzinger complex (32).

In the medulla, the nucleus ambigus and the nucleus of the solitary tract are influenced by both glutamate and dopamine, essential for motor control of respiration. As dopamine levels decline, motor complications arise, affecting respiratory muscles, including the diaphragm (31). These motor signals are transmitted via the phrenic and accessory nerves. Reduced central stimuli from the medulla further impair peripheral responses, leading to increased rigidity, respiratory asynchrony, and dysfunctions in breathing patterns, such as dyspnea and decreased cough reflex. This pathophysiology highlights the critical role of dopamine in maintaining respiratory motor function and the progression of respiratory complications in neurodegenerative conditions (31, 32).

In this sense, the association between gait and respiratory rate is crucial, as overlapping neural circuits regulate both. Evaluating these parameters is essential for detecting early motor-respiratory dysfunctions, which can inform disease progression and therapeutic strategies (13–15), particularly in neurodegenerative conditions where movement and respiratory control are progressively impaired (33). In addition, our findings corroborate those of previous studies with other populations in which the relationship between functional mobility and respiratory function has been well established (13–15). In a review by Guilherme et al. (9), 18 studies with 541 individuals showed a predominance of obstructive, restrictive, and normal patterns across different studies. Our findings indicate that 53% of PwPD with mild to severe disease had impaired respiratory profiles, which is consistent with previous research (34, 35).

This is the first study to associate gait capacity, WOS, and LRI with respiratory profiles in PwPD. Different walking speeds predict respiratory function, and the efficiency of gait, represented by LRIs, reflects expiratory capacity. The study, however, is limited by the lack of an evaluation of postural syndrome, the inability to control past respiratory conditions, and the lack of a classification of PD subtypes. Even so, the study’s clinical application is essential in identifying respiratory disorders associated with gait capacity. SSWS, WOS, and LRI are low-cost and easy-to-apply screening parameters for respiratory changes in patients with moderate Parkinson’s disease. Future research is needed to determine whether walking ability can be a valuable tool for treating respiratory dysfunction in PwPD through personalized locomotor and respiratory rehabilitation.

## Conclusion

PwPD at H&Y stages above 2 is associated with reduced respiratory muscle strength and pulmonary capacity. These outcomes are related to gait capacity, as evidenced by the prediction of the MIP, MEP, FVC, and FEV1/FVC ratio by gait parameters represented by the SSWS, FWS, and LRI. To our knowledge, this relationship represents a crucial clinical factor influenced by reduced functionality and independence in this population. Moreover, gait speed alterations may serve as biomarkers of respiratory changes in PwPD, offering a readily applicable and cost-effective screening tool.

## Data Availability

The datasets presented in this study can be found in online repositories. The names of the repository/repositories and accession number(s) can be found in the article/Supplementary material.
