# Supplementary figures and images for: Can walking capacity predict respiratory functions of people with Parkinson’s disease?
Source: Front Neurol. 2025 Mar 26;16:1531571. doi: 10.3389/fneur.2025.1531571 (PMC11978635; doi:10.3389/fneur.2025.1531571)

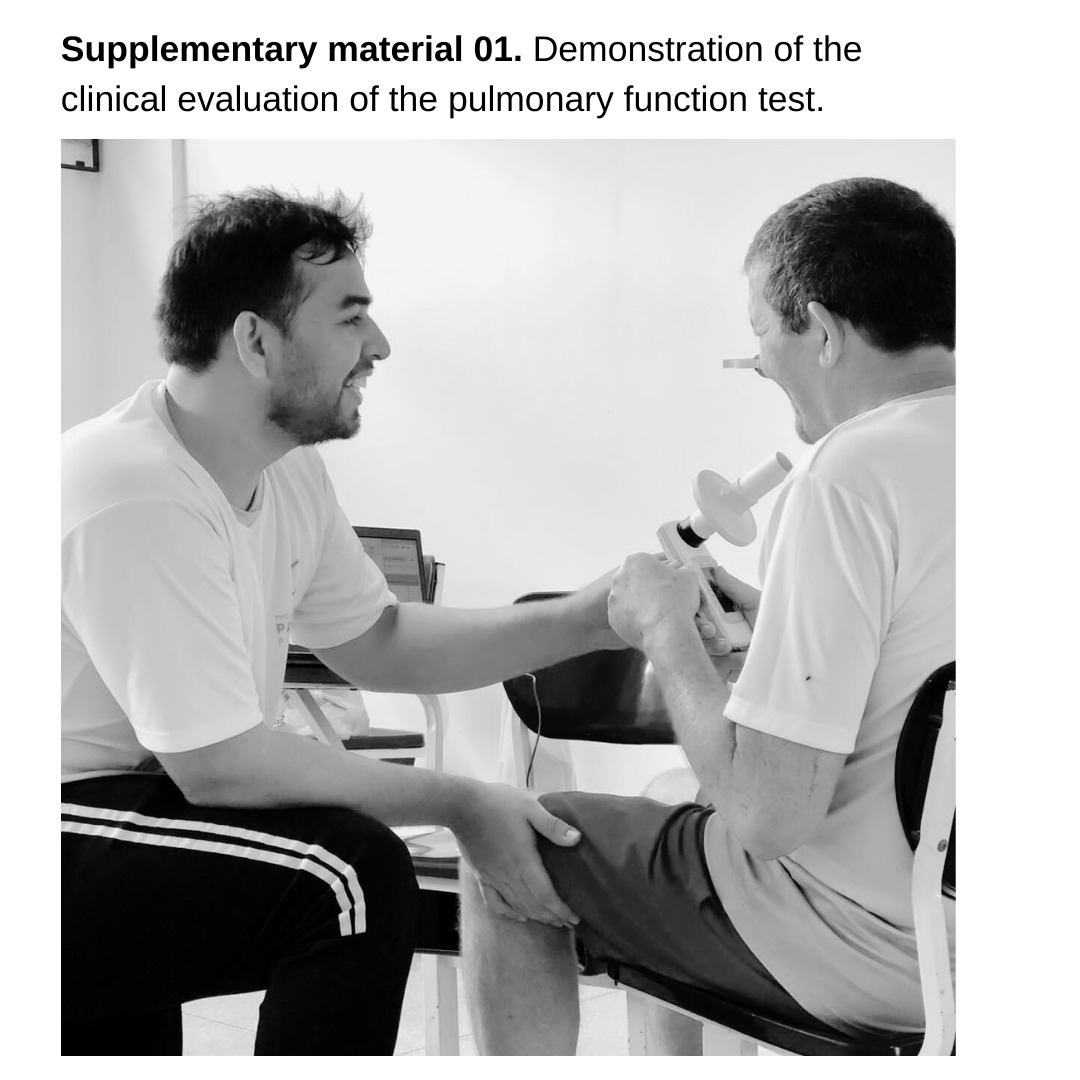

Supplement: Supplementary file 1 [file Image_1.jpeg]

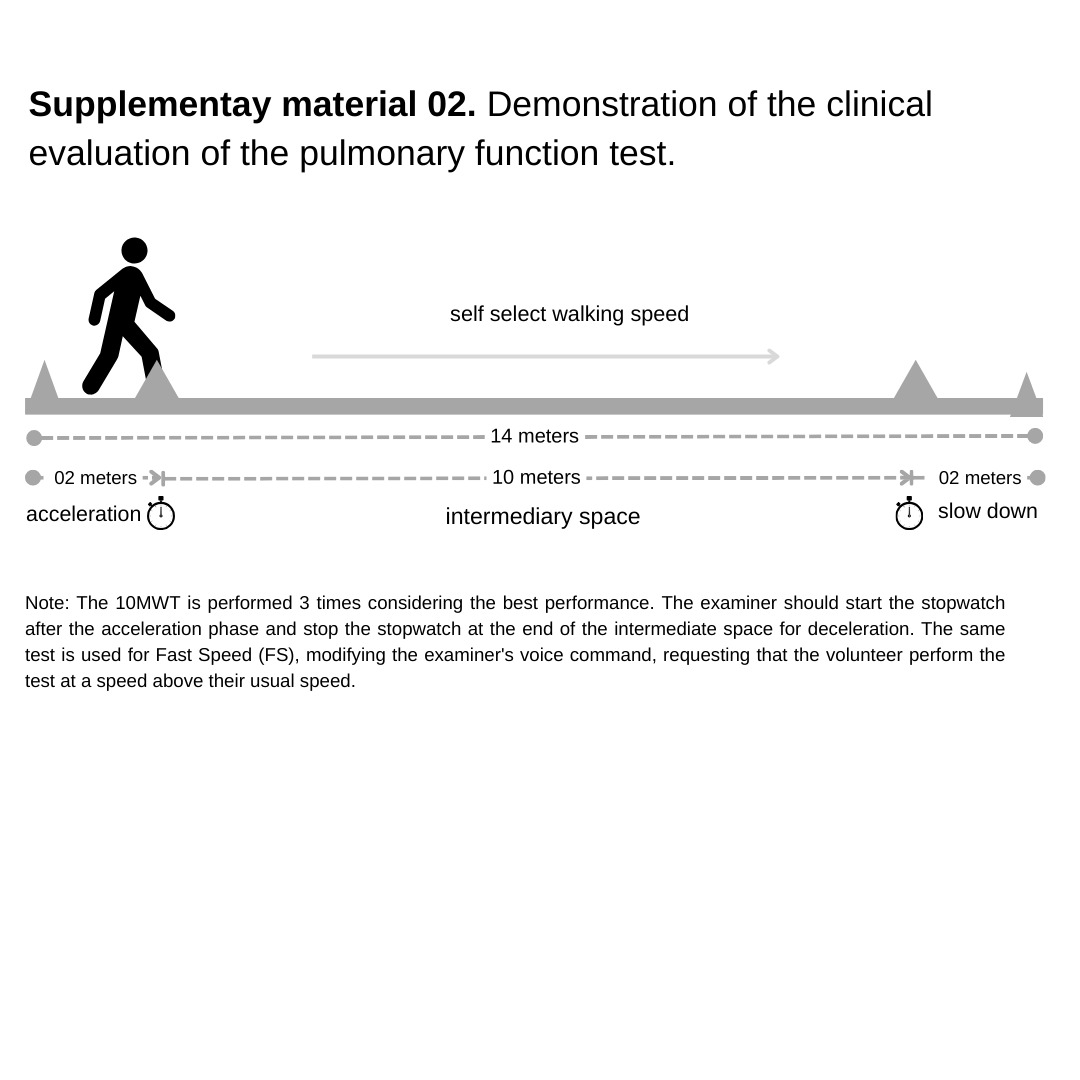

Supplement: Supplementary file 2 [file Image_2.jpeg]
